# Implementation and impact of a social prescribing intervention: an ethnographic exploration

**DOI:** 10.3399/BJGP.2022.0638

**Published:** 2023-07-11

**Authors:** Tessa Pollard, Kate Gibson, Bethan Griffith, Jayne Jeffries, Suzanne Moffatt

**Affiliations:** Department of Anthropology, Durham University, Durham.; Population Health Sciences Institute, Faculty of Medical Sciences, Newcastle University, Newcastle upon Tyne.; Population Health Sciences Institute, Faculty of Medical Sciences, Newcastle University, Newcastle upon Tyne.; Population Health Sciences Institute, Faculty of Medical Sciences, Newcastle University, Newcastle upon Tyne.; Population Health Sciences Institute, Faculty of Medical Sciences, Newcastle University, Newcastle upon Tyne.

**Keywords:** ethnographic methods, health inequalities, health social determinants, link workers, primary health care, social prescribing

## Abstract

**Background:**

Social prescribing involves referral of patients from primary care to link workers, who work with them to access appropriate local voluntary and community sector services.

**Aim:**

To explore how a social prescribing intervention was delivered by link workers and the experiences of those referred to the intervention.

**Design and setting:**

The study used ethnographic methods to conduct a process evaluation of a social prescribing intervention delivered to support those living with long-term conditions in an economically deprived urban area of the North of England.

**Method:**

Participant observation, shadowing, interviews, and focus groups were used to examine the experiences and practices of 20 link workers and 19 clients over a period of 19 months.

**Results:**

Social prescribing provided significant help for some people living with long-term health conditions. However, link workers experienced challenges in embedding social prescribing in an established primary care and voluntary sector landscape. The organisations providing social prescribing drew on broader social discourses emphasising personal responsibility for health, which encouraged a drift towards an approach that emphasised empowerment for lifestyle change more than intensive support. Pressures to complete assessments, required for funding, also encouraged a drift to this lighter-touch approach. A focus on individual responsibility was helpful for some clients but had limited capacity to improve the circumstances or health of those living in the most disadvantaged circumstances.

**Conclusion:**

Careful consideration of how social prescribing is implemented within primary care is required if it is to provide the support needed by those living in disadvantaged circumstances.

## INTRODUCTION

Social prescribing refers to the creation of referral pathways to meet the social needs of patients, often emphasising the referral of patients into community groups and services. It has grown internationally over the past few years^1^^,^^2^ and has emerged as a central plank of the NHS personalised care agenda and long-term plan, which included: *'the aim that over 900 000 people are able to be referred to social prescribing’* by 2024.^3^

NHS England expects social prescribing to reduce GPs’ workload, address the social determinants of health, and reduce health inequalities.^4^^,^^5^

In the UK, social prescribing is generally delivered by link workers, who receive referrals from primary care, discuss social needs with patients, and link them on to community-based services.^1^ NHS social prescribing link workers are expected to: *‘give people time, focusing on “what matters to me”… taking a holistic approach to people’s health and wellbeing.'*^3^ This is in contrast to lighter-touch ‘active signposting’. Social prescribing is also embedded in discourses of choice and empowerment that are central to the personalised care agenda, emphasising the importance of enabling patients to *‘take control’* of their health.^4^^,^^6^ Highlighting these different aspects of social prescribing, Marmot *et al* argue that social prescribing: *‘must include a strong focus on activities to improve the conditions of daily life — through housing and financial advice for example — as well as supporting behaviour change.'*^7^

Although social prescribing has been embraced enthusiastically, little is understood about how it is experienced and whether it works as anticipated.^8^ Process evaluation, including understanding implementation, exploring expected causal mechanisms, and identifying contextual factors affecting outcomes, is increasingly recognised as an essential part of the evaluation of complex interventions,^9^^,^^10^ allowing researchers to *‘open up the black box’* at their heart.^11^

Previous qualitative studies exploring social prescribing have used interviews and focused mainly on professional stakeholders.^12^^–^15 Although qualitative research based on interviews can provide valuable information for process evaluation, it relies on the ability of interviewees to recall and articulate their experiences. Following Bourdieu,^16^ Nettleton and Green^17^ show that much of *‘how and why people act as they do is likely to be beyond their cognitive and rational understanding.'* The current study therefore used an ethnographic approach, based on participant observation along with other qualitative methods, over a period of 19 months, to develop a deep understanding of a social prescribing intervention.^11^ The intervention aimed to support those with long-term conditions and expected, in so doing, to address health inequalities.

**Table:** How this fits in

Social prescribing has been embraced enthusiastically, however, little is understood about whether it works as anticipated. Within one social prescribing intervention it was observed that there was a tension between two approaches to social prescribing, one emphasising intensive support and the other focused on empowering clients to make lifestyle changes. Requirements for link workers to devote time to generating referrals and to focus their efforts on the completion of regular assessments (to generate payments for providers) facilitated a drift towards the latter lighter-touch approach. Although greatly appreciated by some clients, such a lighter-touch approach had limited capacity to improve the circumstances or health of those living with most disadvantage.

Elsewhere the authors have engaged with social theory to examine the intervention from the perspectives of link workers^18^ and clients^19^^,^^20^ separately. Here the authors synthesise both parts of the research to explore how social prescribing was delivered and received, and implications for its ability to meet its stated aims.

## METHOD

### Design and setting

The core method was ‘hanging out’ with service users over extended periods of time, supplemented by shadowing link workers, interviews with link workers and clients, and focus groups with link workers. This work was part of a mixed-methods evaluation of the intervention, which focused on its impact on those with type 2 diabetes^21^ as a common and serious long-term health problem linked to wider social determinants of health.^22^

The intervention was delivered in an ethnically diverse and largely economically deprived urban area of the North of England and quantitative findings showed that it was associated with a small (−0.10 percentage points, 95% CI = −0.17 to −0.03 percentage points) drop in HbA1c for people with type 2 diabetes.^23^

### The social prescribing intervention

The social prescribing service was established 4 years before fieldwork began. It was targeted at people in middle and early older age with at least one of six qualifying long-term conditions. On referral from one of the participating GP practices, patients were assigned a link worker. At their first meeting, the link worker used an assessment instrument to help clients assess their current situation, including ‘lifestyle’ and ‘money’. Based on this assessment a personalised action plan was agreed, following which the link worker was expected to support patients to access relevant local community services, or in some cases, to support them to develop self-directed programmes. ‘Journeys’ with the intervention averaged 18 months but could last for up to 4 years.

At the time of this study the intervention was delivered by two not-for-profit organisations, contracted to an umbrella special purpose vehicle body. This umbrella body held contracts with public sector commissioners and a specialist social investor, pairing an outcomes-based NHS contract with a social impact bond investment. The two providers were given upfront start-up costs, but this approach changed over time to reward successful engagement with clients, and at the time of the current study payments to providers were generated by the completion of the assessment instrument, expected at approximately 6-month intervals. The intervention was a forerunner of the NHS social prescribing programme, and the provider organisations now also deliver NHS social prescribing.

### Participants

Most link workers (*n* = 20) agreed to participate in the study. The authors also engaged with 19 clients of the intervention (‘client’ is the term used by those delivering the intervention), all of whom had type 2 diabetes, usually in combination with other long-term conditions. Clients were purposively sampled to recruit a diverse group across age, gender, ethnicity, employment status, service provider, and time with the intervention.

### Data collection

Participant observation with link workers and clients was conducted, as well as focus groups with link workers and interviews with link workers and clients (Table 1). In addition, if a family member was closely involved with the client’s health, with the client’s permission they were also invited to be interviewed. The fourth author conducted fieldwork with link workers and the second author with clients, each writing detailed fieldnotes. Interviews lasted from 30–120 min and focus groups lasted 90–120 min. All were audio-recorded and transcribed verbatim. Interviews after March 2020 were conducted by telephone because of COVID-19-related social distancing laws. A separate study explored the impact of COVID-19 on the intervention and on clients.^24^^–^26 All link workers and clients who were key participants in this study were given pseudonyms.

**Table 1.** Methods and participants with timings

**Method**	**Participants**	**Purpose**	**Timing**
**Focus groups**	Three groups, total *n* = 16 LWs	Focus groups were used to elicit LWs’ understandings of the aims and implementation of social prescribing	September 2019 to October 2019
**Hanging out in provider organisations’ offices 2 days per week and shadowing LWs in their daily routines**	*n* = 20 LWs, of whom, *n* = 8 individually shadowed	Shadowing was used to gain direct insights into the everyday routines of LWs and their implementation of social prescribing It included attendance at training sessions for LWs	August 2019 to February 2020
**Interviews with LWs**	*n* = 6 LWs	Interviews were used to gain insights from LWs who had not been directly shadowed	October 2019 to June 2020
**Initial interviews with clients**	*n* = 19 clients	Getting to know clients, understanding their personal circumstances and health problems, understanding their interaction with the intervention	January 2019 to June 2019
**Participant observation, including visiting participants’ homes, joining participants in activities such as gardening, the gym and social groups, accompanying clients to meetings with LWs, visiting the foodbank, and so on**	*n* = 19 clients, approximately 200 hours of fieldwork	Gaining a detailed understanding of the ways in which the intervention unfolded in people’s lives	January 2019 to July 2020
**Photo-elicitation interviews**	Subsample of *n* = 9 clients	Photo-elicitation interviews, in which participants were asked to take approximately 10 photographs of health and wellness in their lives, with the photographs subsequently used as prompts in an interview, were used with participants who were less engaged with participant observation	March 2019 to October 2019
**Interviews with family members**	*n* = 7 family members	Interviews with close family members involved in managing the study participants’ health	July 2019 October 2019
**Final interviews with clients, guided partly by intervention data recorded for those clients by LWs**	Subsample of *n* =15 clients	Most participating clients provided final telephone interviews reflecting on their experiences of social prescribing	July 2020

*LW = link worker.*

### Data analysis

The final qualitative datasets comprised interview and focus group transcripts, participants’ photographs, and ethnographic fieldnotes from the two sets of fieldwork. Data analysis was an iterative process, beginning with reflexive fieldnotes and team discussions of themes arising from each ethnography during data collection. When all data had been collected each dataset was analysed separately. For each, a coding framework was developed iteratively during a process of line-by-line coding facilitated by NVivo (version 11).

Memos were used to assist in the process of moving from these content-based descriptive themes to more conceptual themes,^27^ with a focus on answering questions about how social prescribing was implemented, how it worked for clients, and its potential to reduce health inequalities.

Analysis of the link worker data was led by the third author (a GP and PhD student in medical anthropology) and for the client data it was led by the second author (a sociologist), who each met regularly with the first author (a medical anthropologist) and the last author (a social gerontologist). The authors also met regularly as a full team to discuss and compare themes emerging across the two datasets.

The authors documented and refined the themes that cut across the two pieces of fieldwork (which were strikingly similar) in further summaries. The focus was on ‘meshing’ and ‘linking’ the data to explore how different dimensions of context and social processes ‘weave together’^28^ in relation to the intervention. This process allowed the authors to make theoretically driven comparisons across the datasets to generate the overarching analytical themes that are the focus of this paper.

## RESULTS

The data identify challenges in establishing social prescribing as a new service and reveal social prescribing practices as diverse and changeable, shaped partly by understandings of the purpose of social prescribing that differentially emphasised ‘support’ or ‘empowerment’.

A focus on undertaking periodic assessments with clients created by the funding structure also influenced the way in which social prescribing was delivered. Clients themselves had diverse needs and priorities, resulting sometimes in a good and sometimes in a poor ‘fit’ between the intervention and their needs.

### Establishing an identity and place for social prescribing

In line with the expected referral route from primary care, Andy’s GP recommended Andy (see later for further client information) give social prescribing a ‘try’ following a diagnosis with diabetes, and the service was recommended to Anna, who had diabetes, asthma, and other conditions, by a practice nurse. However, others joined social prescribing either through proactively requesting a referral, as in Zaheer’s case, after he saw a poster at his GP practice or, in many instances, via a phone call from a link worker. Only a few of the practices embraced social prescribing, often because of the enthusiasm of an individual member of staff who generated high volumes of referrals.

Each link worker was attached to one or more GP practices, often meeting clients within practice buildings, but in many instances relationships between link workers and primary care staff and structures were weak. Some link workers felt that practice staff lacked understanding or respect for their role and that they were treated as outsiders, going apparently unrecognised by practice staff in corridors and common rooms. Access to information systems and consultation rooms was often restricted, and problematic relationships with practices were a common focus of discussions between link workers and their managers. As a consequence of limited referrals from many practices, link workers became increasingly responsible for recruiting patients into social prescribing. This was a time-consuming and unpopular task that involved telephoning patients meeting referral criteria, a job often referred to unhappily as ‘cold-calling’.

In turn, once link workers had met with clients, navigating a changing landscape of services and groups for onward referral was a continuing challenge for them, with common issues being specific criteria for some services, and long waiting lists. This meant that, despite efforts by the provider and umbrella organisations to keep track of opportunities for onward referrals, and by link workers to establish personal connections, onward referrals tended to follow a few established pathways, with *‘*[advice on] *benefits and the gym’* described by one link worker as the two most common. The fact that other voluntary and community services in the area offered services akin to social prescribing (and over time increasingly also labelled as social prescribing) created tensions that further restricted collaborative working.

Reflecting slow progress towards firmly establishing a place for this social prescribing intervention, some clients had difficulty in distinguishing social prescribing link workers from health professionals or from those working in the voluntary, community and social enterprise (VCSE) sector. Many were unable to recall meeting a link worker or to recognise the term social prescribing. This confusion arose partly because link workers ‘cold-calling’ from GP surgeries often mentioned their affiliation with the surgery to orient the client and establish a clinical legitimacy for their unfamiliar role. For many clients then, social prescribing was not a recognisable service.

Embedding social prescribing within the existing landscape of services was thus challenging, and although some GP practices and onward referral services welcomed the introduction of the intervention, in many cases there was a lack of interest, or sometimes antipathy from members of organisations expected to form part of the referral pathway. This meant that link workers had to spend time generating referrals, establishing their roles, and building relationships, with the knock-on consequence of limiting their time with clients.

### Tensions and heterogeneity in understandings of social prescribing

Brenda, who managed her diabetes without medication, and also had arthritis, was 70 years of age when she joined the current study. When asked why she was interested in getting involved in social prescribing she replied that:

*‘When they told us* [me] *about it they said, like, they can help you with exercises, help you to sort your life out around the diabetes, not the diabetes sorting your life out.’*

(Brenda, Client)

She described how she had had a number of link workers during her time with the service. Her favourite was Dan, who:

*‘Came across like he cared. You know, he made you feel like, when you were there, you were important … he seemed to have more, maybe compassion was the right word I’m looking for.'*

(Brenda, Client)

Dan discussed with Brenda various physical activities that might suit her, responding to her worries about being overtaxed in a Nordic walking group or Zumba classes, and eventually she started doing circuits tailored for her ability at a local community gym.

Shirley had a different experience with social prescribing. She was in her late 50s, worked part-time and had been diagnosed with type 2 diabetes 2 years previously. She told us about her first meeting with her link worker:

*‘It was about 15 minutes, the meeting itself. It was mainly going through diet things, suggestions about what I could* [eat] *for meals. Portion sizes, she went through that … She said, “We’ll get you into the gym”, adding later that “the link worker just talked about exercise and that I needed to exercise for type 2 diabetes.”’*

(Shirley, Client)

Subsequently her link worker phoned to update Shirley on her efforts to set up a gym referral, which bore fruit 6 months later, when Shirley went for her induction. Unfortunately, at that meeting she was told that her blood pressure was too high and referred back to her GP. There was no further contact from her link worker.

Brenda’s description of Dan’s link working suggests that he offered attentive support. Other clients and link workers also described examples of link workers building rapport with clients as well as accompanying clients to activities and services, being in frequent face-to-face contact, and generally being abreast of their ever-changing circumstances.^20^

However, not all link workers emphasised this way of working, and some understood their role as focusing more on motivating and empowering clients to achieve behaviour change, as appears to have been Shirley’s experience. Often this meant encouraging clients to *‘take control’* of their health by (co)creating lifestyle goals, helping them: *‘to feel more empowered to make a difference to their own life and not be as reliant on other people.’* (Abby, link worker [LW]), also facilitating what the authors term ‘unsupported linking’ into local gyms and diet-related services. Interestingly, the assessment instrument encouraged link workers to start assessments by discussing ‘lifestyle’, potentially reinforcing this approach.

According to link workers, in the early days of the intervention different providers took different approaches, with one explicitly offering a ‘behaviour change service’, whereas the other offered more intensive support. By the time of the current fieldwork, both providers were increasingly committed to using behaviour change techniques, such as motivational interviewing. As Marie, a link worker in the provider organisation that had previously focused less on behaviour change, said:

*‘The way that we should work with people has changed over the years. That’s been the hard part because some people* [link workers] *like the handholding and the home visit side of things.’*

(Marie, LW)

Although these two approaches, emphasising either support or empowerment, were not always clearly differentiated nor entirely incompatible, and, as established in the introduction, both are built into the logic of social prescribing, the tension between them surfaced repeatedly through the current fieldwork. For example, some link workers expressed concerns that other link workers sometimes acted as a ‘support worker’, and the term ‘handholding’ was often used (as above) to characterise this way of delivering social prescribing as inappropriate, again emphasising concerns about creating dependency, rather than building responsibility.

Over time, then, there was a drift towards the model of social prescribing that emphasises empowering and motivating clients to take personal responsibility. The authors of the current study suggest that this is a lighter-touch model that also worked better within the time pressures caused by the need for link workers to generate referrals, and alongside a focus on assessment and targets, as described below.

### Assessment and targets

Link workers felt under pressure to prioritise completing assessment instruments with clients, thus generating payments for the provider organisations. The need to complete enough assessments was repeatedly emphasised at link worker training sessions. A list on the wall of one of the shared offices displayed the number of assessments each link worker had completed that month; it was referred to by one link worker as ‘the wall of shame’. Link workers felt pressured to deliver a linear and streamlined intervention that was structured by the need to complete assessments at expected intervals rather than being driven by responsiveness to clients’ needs.

At the time of this fieldwork, some clients felt that they were only contacted for the purpose of completing an assessment, usually over the phone, and several clients described ‘out of the blue’ contacts,^20^ often involving completing an assessment. There was a maximum number of assessments per client that generated payments for providers and some clients, including some who had had long and fruitful social prescribing journeys, experienced being discharged after completing this number, including the participant Zaheer, who was at the time struggling to manage his diabetes and mental health problems in the context of COVID-19.

Some link workers were unhappy with the pressures to work with clients in time-efficient ways that prioritised regular assessments and timely discharge, and sometimes this pressure was actively resisted as link workers sought to do what they considered best for their clients.

As illustrated in these field notes, unhappiness generated by targets contributed to a high turnover in link workers:

*‘LW Amy said “have you seen the photograph in the office? There are only two link workers remaining from that original photograph”. She continues “our role is defined by [assessments], no one is happy. It is not fulfilling … The assessment moves us away from care. All that matters is [assessments].”*

(Author field notes, November 2019)

In turn, high turnover increased caseloads as link workers took on the cases of departed staff, and many clients experienced at least one change of link worker. For one of the participants, Christine (aged 60–64 years, unemployed), this disrupted her experience of social prescribing:

*‘She went for a job higher up, which I was a bit thingied about because I thought I’m just getting used to her.'*

(Christine, Client)

### Classed experiences of social prescribing

The drift towards empowering and motivating had consequences for the impact of social prescribing on inequalities. Class and other forms of inequality shaped clients’ engagement with link workers’ efforts to motivate them to invest in their long-term health; more advantaged clients were more able to engage with the model of social prescribing that had become dominant within the intervention. For example, Andy was a homeowner and graduate with stable employment, and social prescribing gave him a *‘a kick-start, reminder wise, and the memory of what you really should be doing’;* consequently he was able to successfully re-engage with physical activities.

Other clients were not in a position to respond so readily. Carol was brought up by her grandparents before being placed into care, and then experienced domestic violence in her first long-term relationship, subsequently moving into *‘refuge after refuge after refuge.'* When one of the authors met her, she was living in a rented flat after a period of homelessness triggered by problems obtaining benefits. It was from this sanctuary that Carol had begun to address her health, having recently been diagnosed with diabetes, adding to a number of existing health problems. Through her link worker, Carol was referred to a local gym run by a charity, and to healthy eating and smoking cessation classes. However, unlike Andy, Carol was very anxious about going to the gym: *‘when I went the gym, I sobbed my heart out, cried my eyes out.'* Subsequently she was discharged from the intervention because of ‘lost engagement’. At her final interview Carol got extremely upset, recalling how she:

*‘Pushed everybody away, i.e. Amy, that was trying to help me and get me on the right track and everything. And I just couldn’t do it.’*

(Carol, Client)

Carol was keen to engage with social prescribing and with managing her health problems, and did attend the gym for a short period, but partly because of her lack of familiarity with ‘going to the gym’ or, more generally, with investing in her long-term health, and partly because of more immediate concerns, including caring for her sick father, this was very challenging for her.

Andy and Carol then had quite different needs and although catering effectively for Andy by helping him reprioritise physical activity in his life, the intervention was not able to support Carol, nor some other participants in the study living in precarious circumstances for whom taking action to manage their long-term health was both unfamiliar and difficult, given other more immediate priorities.

This distinction between the needs of different clients was recognised by some in the intervention: LW Marie subsequently referred back to her description of the term ‘handholding’ as a problematic approach (see earlier quote), amending what she had said by adding that the aim of the intervention was:

*‘Not reducing handholding, it’s more the staff asking the right questions to identify who actually does really need that support and who could be pushed to do more.'*

(Marie, LW)

However, this distinction was not always pursued by link workers in the face of time constraints that limited their capacity to manage the varying needs of clients.

## DISCUSSION

### Summary

There is no doubt that the social prescribing intervention explored here provided significant help for some people living with long-term health conditions. However, the current analysis highlights structural factors influencing the intervention, which, together with link workers’ understanding, aligned with broader social discourses emphasising personal responsibility for health, encouraged the delivery of social prescribing to drift towards a lighter-touch approach. It was also found that such an approach, although helpful for some clients, had limited capacity to improve the circumstances or health of those living in the most disadvantaged circumstances, and thus to mitigate the social determinants of health or reduce health inequalities.

### Strengths and limitations

The strengths of this study lie in the depth of information obtained using ethnographic methods,^29^ and in combining the perspectives of those delivering and those receiving the intervention. However, other perspectives, such as those of health professionals and those within the VCSE sector, were not included, and nor was it possible to include patients who refused the offer of social prescribing (although clients who had disengaged from the intervention were included).

The authors note that only one model of social prescribing was explored and that the funding model of the intervention differs from that of wider NHS social prescribing.

### Comparison with existing literature

The link worker role was partly shaped by challenges integrating social prescribing between primary care and the VCSE sector, and by the funding structure of the intervention.

Challenges establishing new roles within primary care have previously been observed, including for social prescribing link workers.^13^^,^^30^^,^^31^ Like those in other new roles within healthcare teams, link workers were required to engage in ‘boundary work’^32^ (that is, work to negotiate changes in boundaries between different professions following the introduction of a new role), with primary care and VCSE services, in an effort to develop effective working relationships. They found this work to be time consuming and often dispiriting, partly because of tensions between organisations competing within an underfunded VCSE landscape.^33^ The funding model of the intervention led to further pressures on link worker time and affected the timing and content of interactions with clients in adverse ways. Similar payment structures, including those defined by social impact bonds, have previously been observed to have perverse effects on the delivery of services, both within health care and the third sector.^34^^,^^35^ A consequence of these changes to the anticipated model of link working was a high turnover of link workers, further limiting capacity to develop relationships with primary care, the VCSE, and clients.

The tension observed in the current study between divergent understandings of social prescribing within the intervention reflects diverse understandings of social prescribing within policy discourses and the scientific literature.^6^^,^^13^ A drift over time within interventions to an understanding that shifts responsibility onto individuals targeted by interventions has previously been identified^31^^,^^36^ and has been attributed partly to the pressures of targets and workload.^37^ Such drift, and associated ‘citizen shift’,^36^ is also a reflection of dominant discourses that construct individuals as capable of exercising autonomy and personal responsibility.^12^ Pursuing a ‘healthy lifestyle’ is then seen as the responsibility of individuals,^38^ whatever their circumstances, and this perspective inevitably plays out in the operating practices of organisations and in the approaches of the professionals implementing interventions.^36^

Mackenzie *et al*^37^ persuasively argue that interventions that target the behaviours of individuals in this way have very limited capacity to address structural determinants of health. The more responsibilities for making changes are passed on to clients, the less effective such interventions can be in reducing inequalities because those in the most disadvantaged positions are least likely to benefit.

The empirical data in the current study confirm that Brown *et al*^39^ were right to worry that: *‘even if social prescribing is effective for some, it may fail to help those most in need, and it could exacerbate existing inequalities.'*

### Implications for practice

In conclusion, the findings from this study highlight a need for greater consideration of how social prescribing should operate. If it is to ameliorate inequalities it will need to prioritise a supportive mode of delivery^37^^,^^40^ and give link workers the time to offer such support.

Care is needed to integrate social prescribing with primary care and the VCSE sector, to limit link workers’ caseloads, and to ensure that output or outcome measures do not distort delivery.

In addition, even if these issues can be addressed, there are huge challenges in trying to address structurally derived health inequalities through an individualised approach in the context of underfunded health and VCSE services.
